# Increased sodium fluorescein transport by corticosteroids is inhibited by a LAT-1 specific inhibitor in retinal pigment epithelial cells in vitro

**DOI:** 10.1038/s41598-023-50196-z

**Published:** 2023-12-27

**Authors:** Norihiko Misawa, Shigeru Honda

**Affiliations:** Department of Ophthalmology and Visual Sciences, Osaka Metropolitan University Graduate School of Medicine, 1-4-3 Asahi-machi, Abeno-ku, Osaka, 545-8585 Japan

**Keywords:** Molecular medicine, Pathogenesis

## Abstract

To investigate whether aldosterone (ALD) and hydrocortisone (HC) change the gene expression of *SLC7A5,* which encodes the large neutral amino acid transporter small subunit 1 (LAT1), and the transport activity of LAT1 in the retinal pigment epithelium (RPE) in vitro. ARPE-19 cells were grown to confluence. After withdrawing the serum, ALD or HC was added with several doses and incubated, and *SLC7A5* gene expression was measured. The influx and efflux transport of sodium fluorescein (Na-F) were evaluated using the Transwell culture system. *SLC7A5* gene expression was upregulated by ALD and downregulated by HC in a dose-dependent manner. Both ALD and HC significantly increased the influx and efflux Na-F transport of RPE cells at a dose that did not change the expression of *SLC7A5*. JPH203, a specific inhibitor of LAT1, significantly reduced accelerated Na-F transport. Both ALD and HC increased the gene expression of zonula occludin-1 (ZO-1) although they did not change the immunoreactivity of ZO-1 in RPE cells. LAT1 may play an important role in increasing Na-F transport associated with ALD and HC administration. A specific LAT1 inhibitor may effectively regulate the increased material transport of RPE induced by ALD and HC.

## Introduction

Central serous chorioretinopathy (CSC) is a common retinal disorder that often affects the vision of middle-aged people^1,2^. The clinical characteristics of CSC are well described and consist of serous retinal detachment accompanied by focal or diffuse dye leakage into the subretinal space as determined by fluorescein angiography^3,4^. Although the molecular pathogenesis of this disease is poorly understood, several studies have demonstrated an association between steroid hormones, including external use of systemic or local corticosteroids, and the incidence of CSC^4–8^. However, the mechanism of CSC associated with corticosteroids has not been disclosed.

We recently conducted a two-stage genome-wide association study (GWAS) and found that *SLC7A5* in chromosome 16q24.2 is a possible CSC-susceptible gene^9^. *SLC7A5* codes for the large neutral amino acid transporter small subunit 1 (LAT1), one of the major System L amino acid transporters that mediate the transport of large neutral amino acids with branched or aromatic side chains in a Na + -independent manner^10^. LAT1 is predominantly expressed in the brain, placenta, and testis^11^. In the eye, LAT1 is expressed in the retinal pigment epithelium (RPE)^12,13^, retinal vascular endothelial cells^14–18^, Müller cells^19^, and ciliary non-pigmented epithelium^20^. In polarized epithelial and endothelial cells, LAT1 is considered to play an important role in the transportation of various neutral amino acids at the plasma membrane^21^, but the actual involvement of LAT1 in the pathogenesis of CSC remains unknown.

In this study, we investigated *SLC7A5* gene expression in cultured RPE and evaluated its alteration with corticosteroids. In addition, we observed a change in the transport activity for sodium fluorescein (Na-F) in RPE incubated with corticosteroids and evaluated the effect of a LAT1-specific inhibitor on transport activity*.*

## Results

### Cell viability after corticosteroid treatment

**Figure 1:**
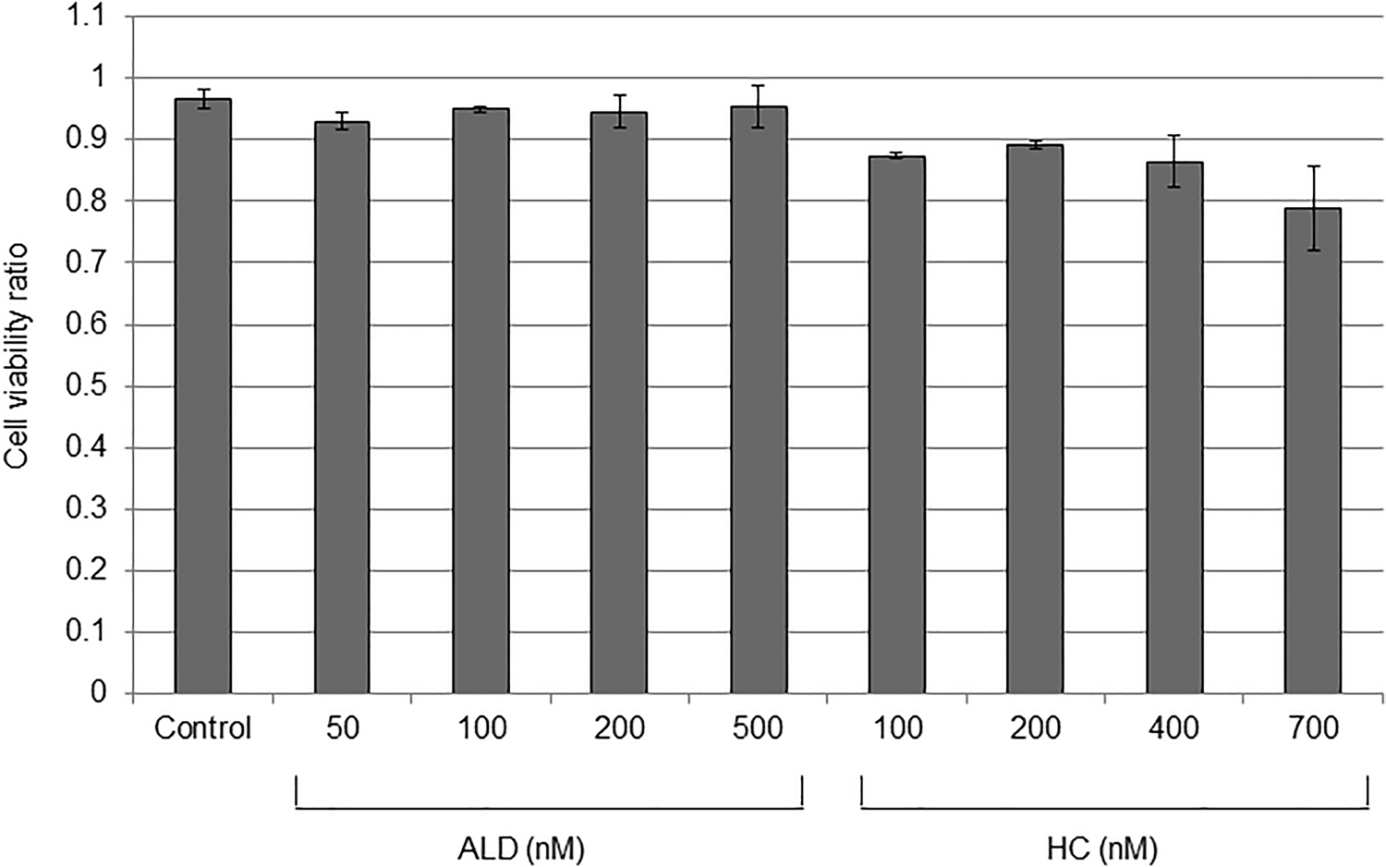
The viability of ARPE-19 cells with several doses of aldosterone (ALD) and hydrocortisone (HC). Cells were seeded at 600,000 cells/cm^2^ and maintained at 37 °C in 5% CO_2_ for 14 days to achieve a confluent state. The serum was withdrawn and cells were further incubated with or without aldosterone (ALD) or hydrocortisone (HC) for 12 h. Cell viability was estimated by the exclusion of 0.4% trypan blue solution. The number of living cells was determined using a hemocytometer. To ensure reproducibility, experiments were repeated three times. Values are presented as means ± SEMs.

The established RPE cell line ARPE-19 was used in this study. Cells were seeded at 600,000 cells/cm^2^ and maintained at 37 °C in 5% CO_2_ for 14 days to achieve a confluent state. Serum was withdrawn and cells were further incubated with or without several doses of aldosterone (ALD) or hydrocortisone (HC) for 12 h. There was no difference in cell viability among the control, ALD, and HC groups under the experimental conditions in the present study (Fig. 1). The LAT1-specific inhibitor JPH203^22^ at 10 µM of concentration did not affect the viability of cells (data not shown).

### Changes in *SLC7A5* expression by siRNA

*SLC7A5* gene expression was suppressed by siRNA in a dose-dependent manner and reached a plateau with 5 pmol or more of siRNA (suppl. Fig. 1). Following this result, 5 pmol of siRNA was used to inhibit *SLC7A5* in the Na-F transport assay.

### In vitro *SLC7A5* gene expression after corticosteroid treatment

**Figure 2:**
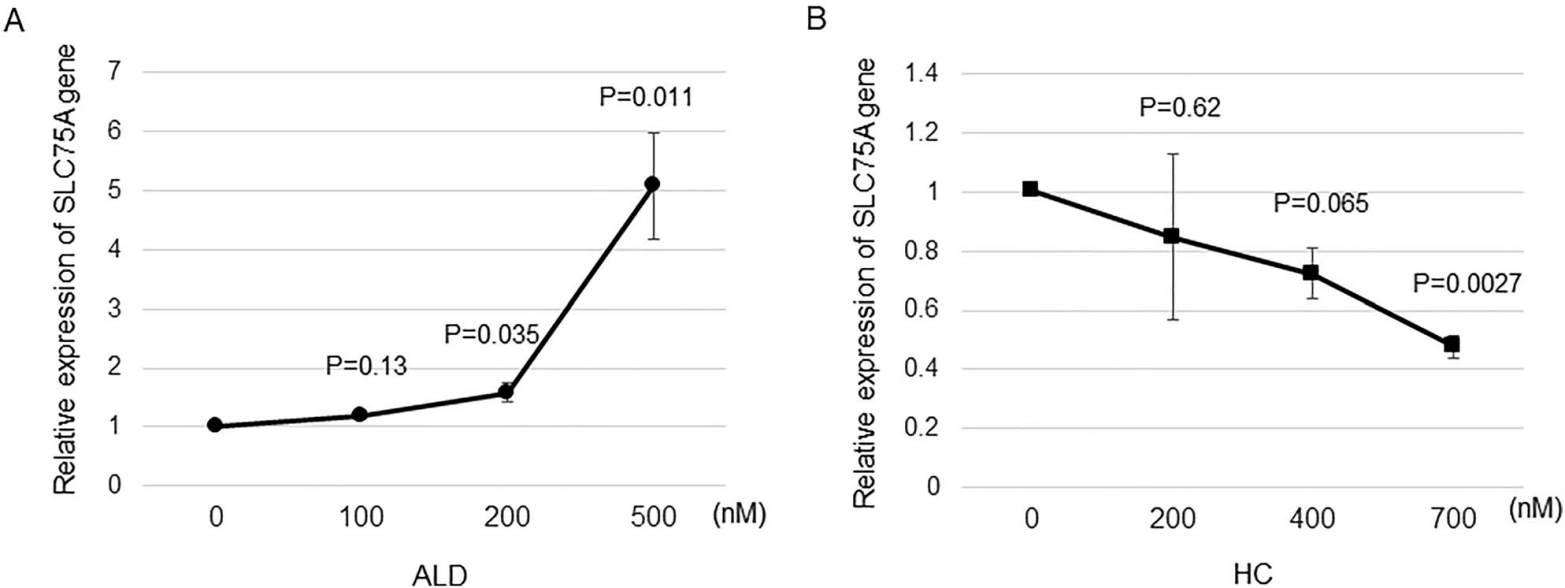
The expression of the *SLC7A5* gene in ARPE-19 cells cultured with (**A**) ALD or (**B**) HC. Cells were grown to achieve a confluent state. The serum was withdrawn and cells were further incubated with or without ALD for 12 h. The real-time PCRs were performed. The amount of targeted gene expressed was normalized to an endogenous reference, *GAPDH*. All measurements were repeated three times and all experiments were conducted in triplicate. Values are presented as means ± SEMs.

*SLC7A5* gene expression was upregulated by ALD in a dose-dependent manner. It was significant at 200 nM or higher concentrations (Fig. 2A). In contrast, SLC7A5 gene expression was downregulated by HC in a dose-dependent manner. It was significant at 700 nM (Fig. 2B). JPH203 at 10 µM did not change *SLC7A5* gene expression (suppl. Fig. 2). Following these results, fluorescein transport assays were conducted with 100 nM of ALD and 400 nM of HC, which were the maximum concentrations not induce the alteration in the gene expression of *SLC7A5* to avoid the possible influence of gene expression change on the amount of Na-F transport associated with LAT1.

### Changes in sodium fluorescein transport by corticosteroid treatment

**Figure 3:**
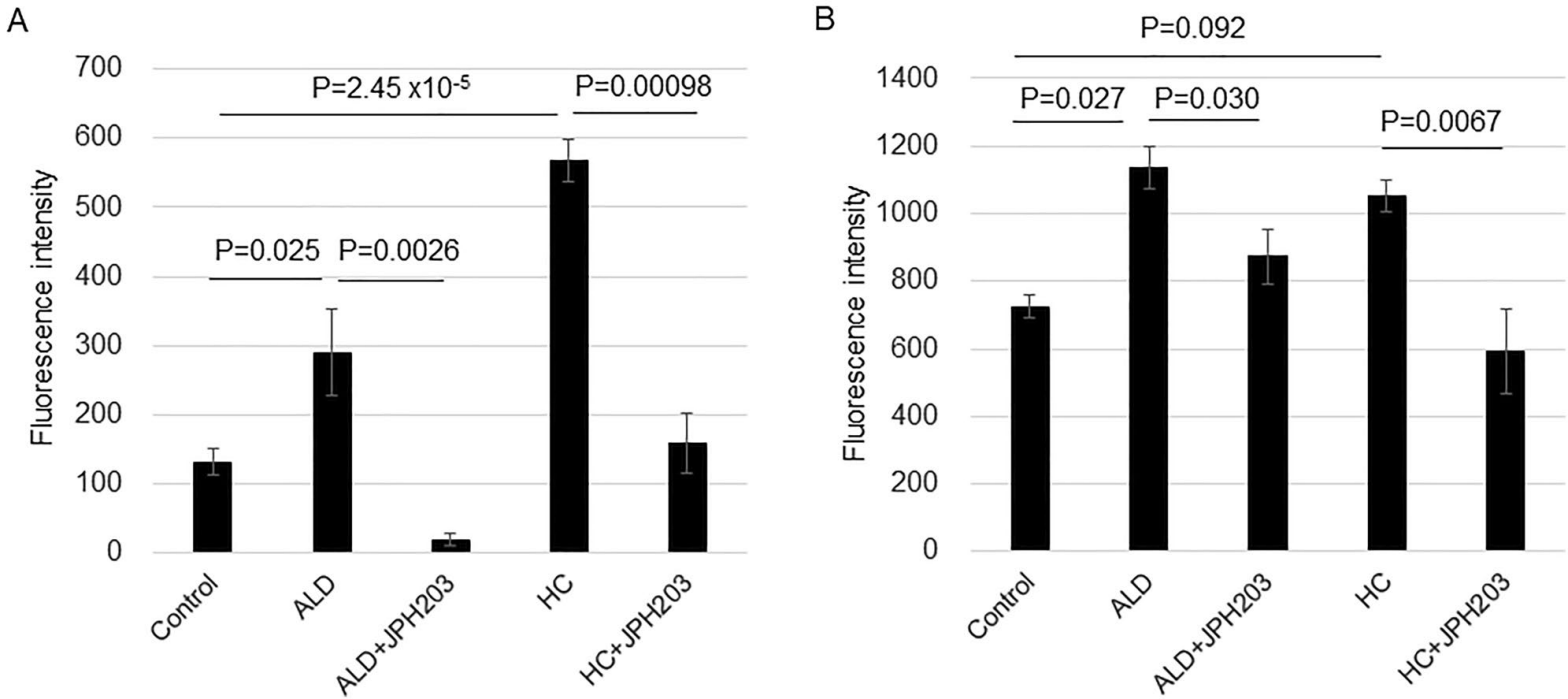
The transport of sodium fluorescein (Na-F) of ARPE-19 cells. (**A**) From the basal side to the apical side (influx transport). (**B**) From the apical side to the basal side (efflux transport). Cells were grown in culture inserts to be confluent. The serum was withdrawn and cells were further incubated with ALD (100 nM) or HC (400 nM) for 8 h. For pharmacological LAT1 inhibition, 10 µM of JPH203 was added. After incubation, Na-F was added to the (**A**) lower or (**B**) upper chamber at 10 µg/ml. After 30 min of incubation, fluorescence in the (**A**) upper or (**B**) lower chamber was measured. Values are presented as means ± SEMs of triplicate.

**Figure 4:**
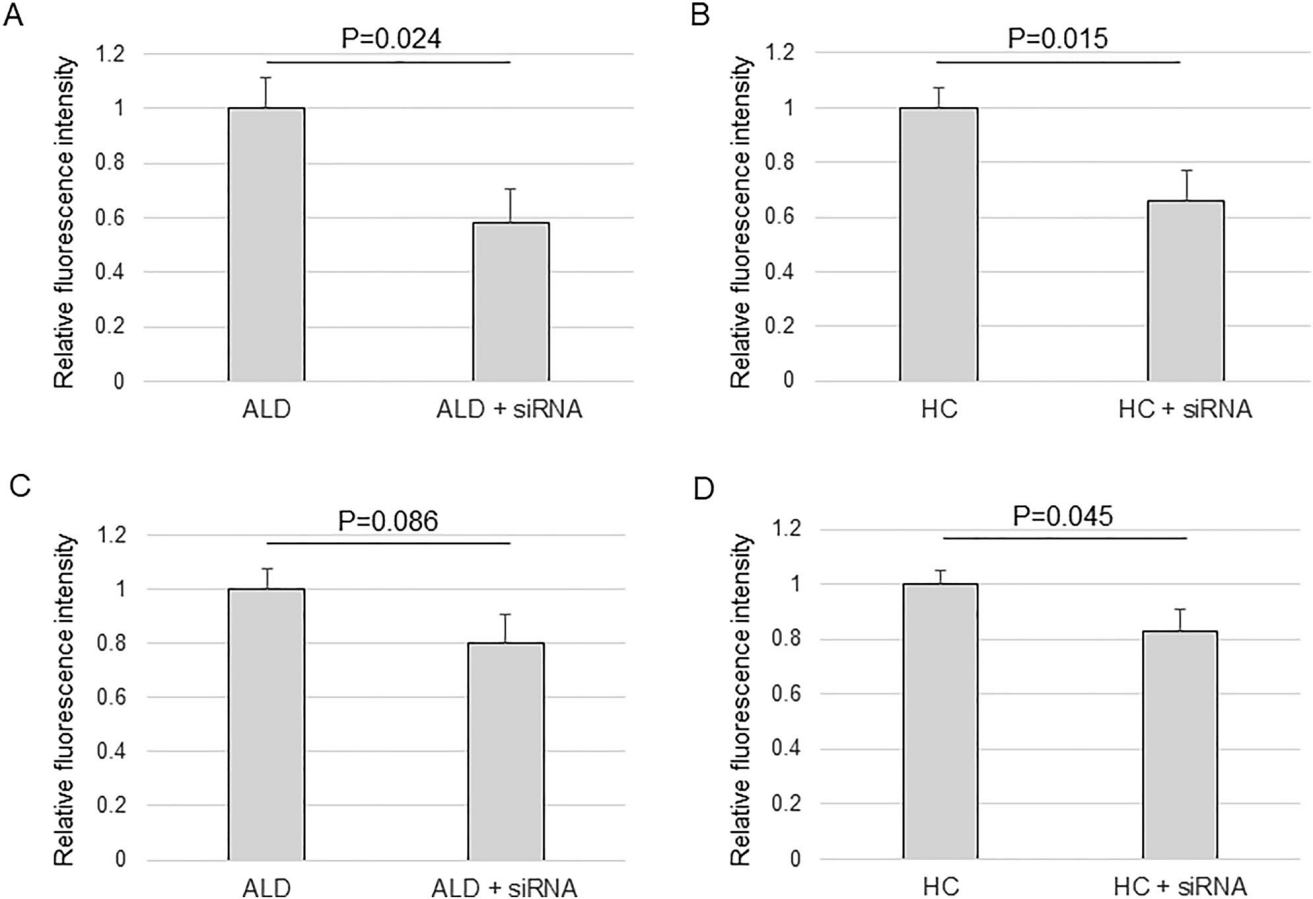
The relative inhibition in the transport of sodium fluorescein (Na-F) of ARPE-19 cells treated by siRNA for *SLC7A5*. (**A**,**B**) influx transport. (**C**,**D**) efflux transport. Cells were grown in culture inserts to be confluent. The serum was withdrawn and cells were incubated with 5 pmol of siRNA for 24 h to knock down *SLC7A5*. The cells were further incubated with ALD (100 nM) or HC (400 nM) for 8 h. After incubation, Na-F was added to the (**A**,**B**) lower or (**C**,**D**) upper chamber at 10 µg/ml. After 30 min of incubation, fluorescence in the (**A**,**B**) upper or (**C**,**D**) lower chamber was measured. Values are presented as means ± SEMs (n = 8).

The baseline transport of Na-F from the apical side to the basal side (efflux transport) was greater than that from the basal side to the apical side (influx transport) (Fig. 3A,B). The influx transport was significantly increased with ALD and HC by 2.2 and 4.3 times, respectively, compared with the control (Fig. 3A), and these transports were significantly inhibited by JPH203. The increase in efflux transport was 1.6 times and 1.5 times with ALD and HC, respectively, compared with the control, and the change was significant with ALD (Fig. 3B). These transports were significantly inhibited by JPH203. The siRNA for *SLC7A5* also significantly inhibited influx transports of Na-F induced by ALD and HC (Fig. 4A,B) and an efflux transport induced by HC (Fig. 4D), but not for an efflux transport induced by ALD (Fig. 4C). A negative control siRNA did not show these inhibitions.

### Expression of zonula occludens-1 (ZO-1) and occludin (OCLN) after corticosteroid treatment

**Figure 5:**
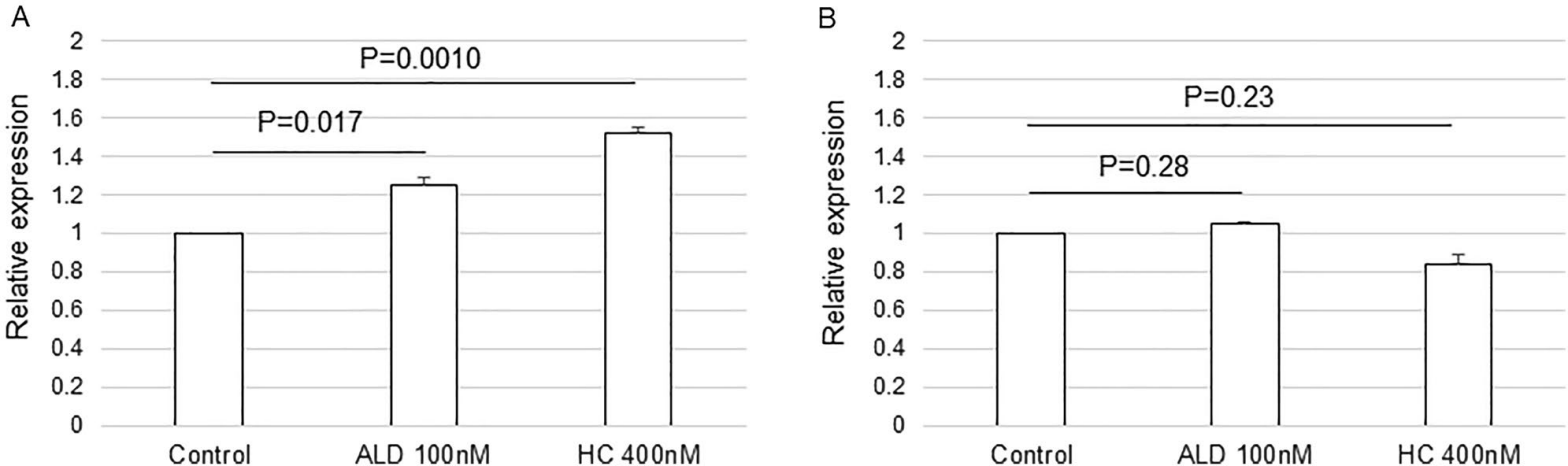
The gene expression of tight junction components in ARPE-19 cells cultured with ALD (100 nM) or HC (400 nM). (**A**) The expression of *ZO-1*. (**B**) The expression of *OCLN*. Cells were grown to achieve a confluent state. The serum was withdrawn and cells were further incubated with or without ALD or HC for 12 h. The real-time PCRs were performed. The amount of targeted gene expressed was normalized to an endogenous reference, *GAPDH*. All measurements were repeated three times and all experiments were conducted in triplicate. Values are presented as means ± SEMs.

**Figure 6:**
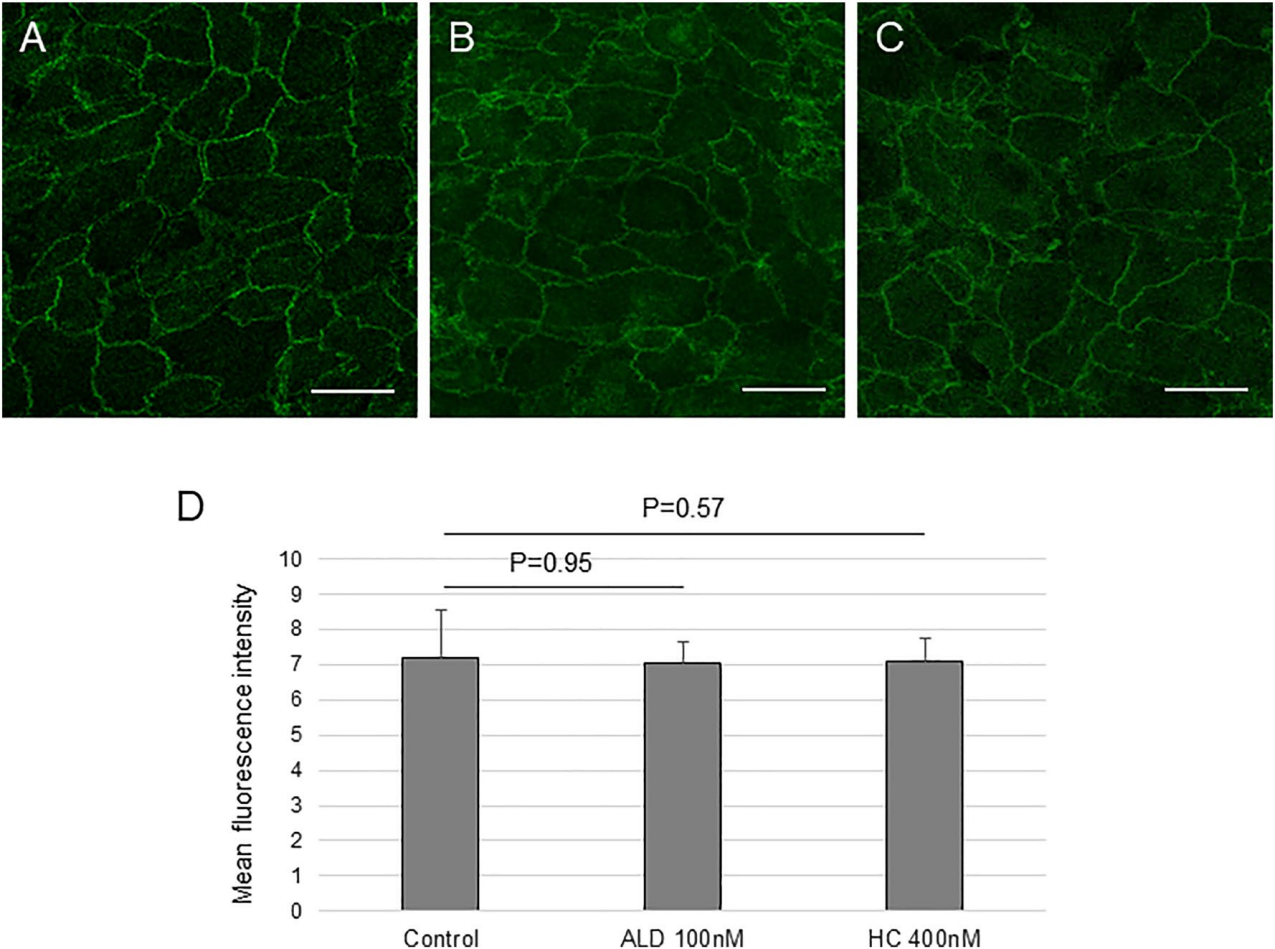
The immunostaining of ZO-1 in the ARPE-19 cells. Cells were grown to reach confluent. Serum was withdrawn and cells were further incubated with or without ALD (100 nM) or HC (400 nM) for 8 h. The cells were fixed with 10% paraformaldehyde and were incubated in rabbit anti-human ZO-1 antisera. The immunoreactivity for ZO-1 was observed with a confocal laser scanning microscope. The fluorescence intensity of the cells was measured using ImageJ software and the mean intensity was calculated from three different areas of glass slide in each sample. The experiments were conducted in triplicate. (**A**) Control. (**B**) Treated with ALD. (**C**) Treated with HC. Scale bar: 20 μm. (**D**) The mean fluorescence intensity from the cells cultured in control, ALD or HC.

To evaluate whether 100 nM of ALD (≒3.6 μg/dl) and 400 nM (≒14.5 μg/dl) of HC used in the Na-F transport assay affect the expression of tight junction components or not since it makes the outer blood-retinal barrier against passive Na-F penetration through the paracellular pathway. Both ALD and HC significantly increased the gene expression of *ZO-1* (Fig. 5A), but did not affect the expression of *OCLN* (Fig. 5B). Immunocytochemistry showed the immunoreactions of ZO-1 around the cells, which indicated its distribution at the plasma membrane of ARPE19 cells (Fig. 6A–C). The appearance of immunoreactions in the cells incubated with 100 nM of ALD (Fig. 6B) or 400 nM of HC (Fig. 6C) was similar to that in the control (Fig. 6A). The mean fluorescence intensity from the cells was not different among the control, ALD, and HC groups (Fig. 6D).

## Discussion

In the present study, we have demonstrated for the first time that ALD and HC increase the Na-F transport of ARPE-19 cells without a change in LAT1 expression in vitro and that a specific inhibitor of LAT1 significantly reduces the transport.

LAT1 is located in the plasma membrane of RPE cells^12,13^. LAT1 plays an important role in the transport of amino acids, bioactive substances such as L-dopa, and several drugs^21,23^. Although there is no report investigating the effects of corticosteroids on the gene or protein expression of LAT1 in RPE, we found that *SLC7A5* expression was differently regulated between ALD and HC, i.e., ALD increased and HC decreased *SLC7A5* expression in a dose-dependent manner. A previous report demonstrated that dexamethasone, a synthetic glucocorticoid, downregulated *SLC7A5* expression in a human placental choriocarcinoma cell line^24^. Another report showed increased protein expression of LAT1 in jejunal epithelial cell membranes from aldosterone-treated rats^25^. Although we used a different cell line, the results of the present study are likely consistent with those of previous reports. The mechanisms by which ALD and HC regulate the expression of *SLC7A5* differently are unclear, but the different affinity of mineralocorticoid receptor (MR) and glucocorticoid receptor (GCR) for ALD and HC could be associated with this phenomenon^26^. The GCR exclusively binds to glucocorticoids, whereas the MR binds to both ALD and glucocorticoids equally, which might influence subsequent intracellular gene profiles and alter *SLC7A5* expression. Further investigations will be required to determine the detailed molecular mechanisms that regulate the expression of *SLC7A5*.

In the fluorescein transport assay, the greater baseline efflux transport of Na-F than influx transport in ARPE-19 cells was consistent with a previous report^27^. To our knowledge, this is the first report that showed that both ALD and HC increased the influx and the efflux transport of Na-F in RPE cells in vitro. In addition, it was notable that increases in influx and efflux transport were significantly inhibited by JPH203, a specific LAT1 inhibitor^22^. This suggests that LAT1 plays an important role in the Na-F transport of ARPE-19 cells. In addition, ALD and HC increased the influx of Na-F transport more than the efflux transport. We speculated that this was because LAT1 is predominantly expressed on the apical side of RPE^28^. The increased Na-F transport with ALD and HC may be modestly inhibited by siRNA for *SLC7A5* compared with JPH203 because siRNA only suppressed newly expressed *SLC7A5* and pre-existing LAT1 was not inhibited by siRNA. However, it remains unknown whether Na-F was transported through LAT1 itself or through other pathways associated with LAT1. In the present study, ALD and HC significantly increased the gene expression of *ZO-1*, coding a well-known tight junction-associated protein and adherens junction protein, which could affect the Na-F transport. A previous report demonstrated increased expression of ZO-1, occludin, and claudin-5 by glucocorticoids in primary bovine retinal endothelial cells^29^. Although immunohistochemistry did not reflect the increased gene expression of *ZO-1* in this study, the integrity of the tight junction is an important factor in paracellular transport. Because an increase in the expression of tight junction proteins is considered to strengthen the tight junction, which likely results in a decrease in the paracellular transport of Na-F, the increase in Na-F transport by ALD and HC was unlikely to be associated with the paracellular pathway. Moreover, the mechanisms by which ALD and HC increase LAT1-associated Na-F transport without changing its expression should be investigated. Although Na-F could be a substrate of LAT1, a previous report demonstrated that non-selective cation channel activity was upregulated by co-expression of 4F2hc/LAT1 with serum/glucocorticoid-regulated kinase 1 (SGK-1), a serine/threonine kinase of the MR downstream pathway, in Xenopus oocytes^30^. Because ALD and HC show equivalent binding affinities to MR^25^, both steroids may have a similar effect on the membrane transport activity associated with LAT1.

The limitation of this study is that all results were obtained from the experiments in vitro. Although we evaluated the outer blood-retinal barrier integrity by the expression of tight junction-associated proteins, it would be better to measure the transmembrane resistance to assess the actual barrier function. Whether ALD and HC increase the Na-F transport associated with LAT1 and if JPH203 inhibits the Na-F transport induced by ALD and HC should be confirmed in vivo. However, the present study indicated a possible association of LAT1 with Na-F transport in ARPE-19 cells, which may simulate the diffuse subretinal leakage of Na-F found in some CSC cases. A specific LAT1 inhibitor may effectively regulate the increased material transport of RPE induced by ALD and HC, which could be a therapeutic modality for CSC.

## Methods

### Materials

ARPE-19 cells were kindly provided by the Department of Ophthalmology, Nara Medical University (Nara, Japan). ALD and HC were purchased from Sigma-Aldrich (Saint Louis, MO, USA). JPH203, a specific LAT1 inhibitor, was kindly provided by J-Pharma Co., Ltd., (Kanagawa, Japan).

### Cell cultures

The routine maintenance of the human RPE cell line ARPE-19 was described previously^31^. Cells were seeded at 600,000 cells/cm^2^ in each 25-mm^2^ flask with Dulbecco’s Modified Eagle medium/Nutrient mixture F12 with 15 mM Hepes buffer (DMEM/F12; Sigma-Aldrich) + 10% fetal bovine serum (FBS; Biosera, Nuaille, France), 2 mM l-glutamine solution (Sigma-Aldrich), and kept at 37 °C in 5% CO_2_ for 14 days to achieve a confluent state. Serum was withdrawn, and cells were further incubated with or without ALD or HC for 12 h, followed by cell viability assay and gene expression experiments.

### Cell viability assessment

Cells were washed twice in Dulbecco’s phosphate-buffered saline (DPBS) (Sigma-Aldrich) and harvested with 0.5%trypsin/EDTA solutions. Cell viability was estimated by the exclusion of 0.4% trypan blue solution (FUJIFILM Wako, Osaka, Japan). The number of living cells was determined using a hemocytometer. To ensure reproducibility, the experiments were repeated three times.

### RNA extraction

Total RNA was extracted from cells using the RNeasy Plus Mini-kit (Qiagen, Valencia, CA) according to the manufacturer’s instructions. Total RNA was eluted from the column in 50 µl RNase-free water. The purity and concentration of RNA were determined by measuring the absorbance at 260 and 280 nm.

### Real-time quantitative PCR

Total RNA was reverse-transcribed into cDNA in a total volume of 100 µl using random hexamers as primers using the Senscript RT Kit (Qiagen) according to the manufacturer’s instructions. Real-time PCRs were performed in 48-well plates on a StepOne™ Real-Time PCR System (Applied Biosystems, Foster City, CA) using dilutions of first-strand cDNA with a final concentration of 1 × Assays-On-Demand (Applied Biosystems) and 1 × TaqMan Universal PCR Master Mix (Applied Biosystems). The assay IDs of the TaqMan probes used in this study were SLC7A5 (Hs01001189_m1), ZO-1 (Hs01551871_m1), OCLN (Hs05465837_g1), and GAPDH (Hs04420697_g1). Each sample was analyzed three times. The thermal cycler conditions were: 2 min at 50 °C, 10 min at 95 °C, followed by 40 cycles of 15 s at 95 °C and 1 min at 60 °C. The amount of targeted gene expressed was normalized to an endogenous reference, *glyceraldehyde-3-phosphate dehydrogenase* (*GAPDH*), and was determined relative to a control using the ΔΔthreshold cycle (CT) method^32^. The target gene CT and endogenous reference CT were calculated for each sample. CT of the endogenous reference was then subtracted from CT of the target gene. This value is known as ΔCT. The ΔCT of each sample was then subtracted from the ΔCT of the control, and this value is known as ΔΔCT. All measurements were repeated three times and all experiments were conducted in triplicate.

### RNA interference

For silencing *SLC7A5*, Silencer® Select (catalog #4392420, assay ID s15653), which included a pre-designed siRNA for *SLC7A5*, was obtained from Thermo Fisher Scientific (Tokyo, Japan). The siRNA was transfected into the culture cells using lipofectamine RNAiMAX® (Thermo Fisher Scientific) according to the manufacturer’s instructions. Silencer® Select negative control No.1 siRNA (catalog #4390843, Thermo Fisher Scientific) was also applied to examine off-target effects. Cells were either transfected or untransfected with siRNA and cultured for 24 h before the experiments. The experiments were repeated 3–5 times.

### Fluorescein transport assay

**Figure 7:**
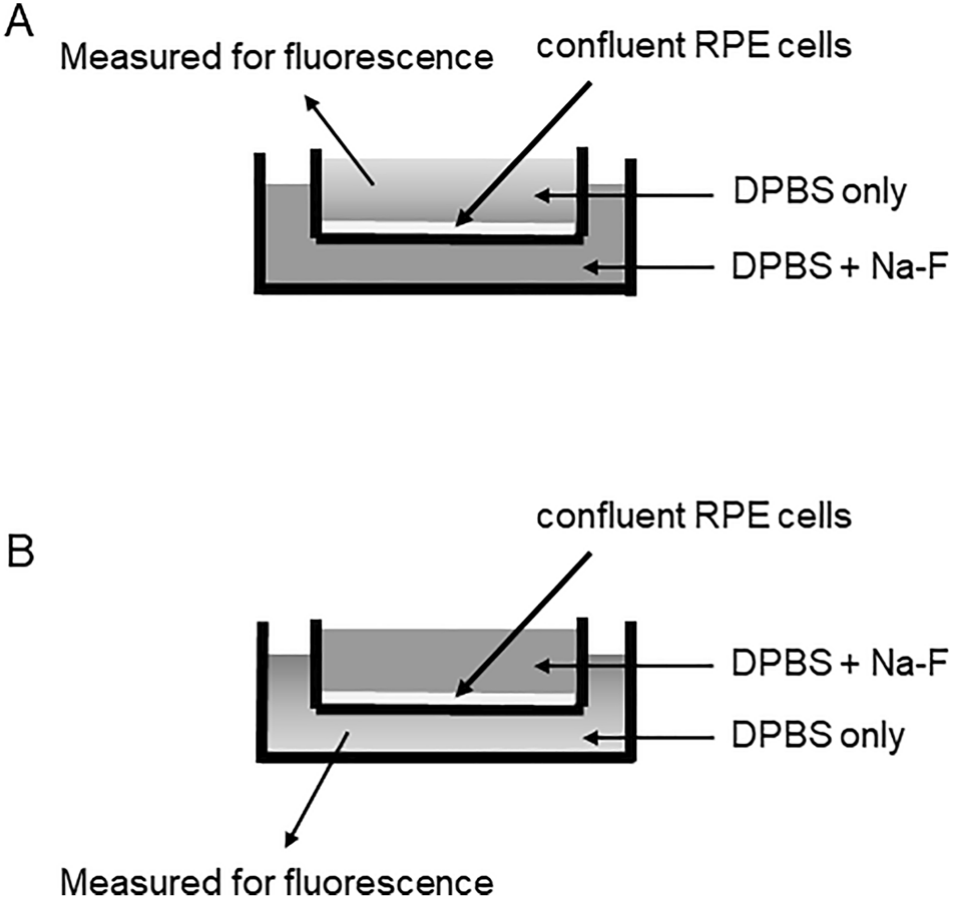
Scheme of fluorescein transport assay. Sodium fluorescein was added to (**A**) the lower chamber or (**B**) the upper chamber at 10 μg/ml. After 30 min of incubation, fluorescence in the upper or lower chamber was measured. *RPE* Retinal pigment epithelium, *DPBS* Dulbecco’s phosphate-buffered saline, *Na-F* Sodium fluorescein.

Cells were cultured in culture inserts (polycarbonate membrane, 0.4 µm pore size, 1.2 cm^2^ surface, Transwell ®, Corning Costar, USA) for at least 14 days until confluence. Serum was withdrawn and cells were further incubated with ALD (100 nM) or HC (400 nM) for 8 h. For pharmacological LAT1 inhibition, 10 µM of JPH203 was dissolved in water and added during the incubation for 8 h according to previous reports^22^. After incubation, the upper and lower chambers were washed twice with DPBS, and 1 ml of new DPBS without ALD, HC, or JPH203 was supplied to both chambers. Sodium fluorescein (Na-F) (Novartis, Basel, Switzerland) was then added to the lower chamber (Fig. 7A) or the upper chamber (Fig. 7B) at 10 µg/ml. After 30 min of incubation, fluorescence in the upper or lower chamber was measured by excitation using a Varioskan LUX™ multimode microplate reader (Thermo Fisher Scientific, Tokyo, Japan). The excitation wavelength was 485 nm and the absorption wavelength was 535 nm. Each sample was measured three times, and all experiments were conducted in triplicate.

### Immunohistochemistry

ARPE-19 cells were grown on an 8-well slide and chamber (WATSON Bio Lab, Tokyo, Japan) to reach confluence. Serum was withdrawn and cells were further incubated with or without 100 nM ALD or 400 nM HC for 8 h. The cells were fixed with 10% paraformaldehyde for 5 min, rinsed in 0.1% phosphate-buffered saline with Tween (PBST), and blocked with 1% bovine serum albumin (#019–27,051, FUJIFILM Wako Pure Chemical Corporation, Osaka, Japan) for 30 min at room temperature. They were incubated in PBST with 1:200 rabbit anti-human zonula occludens-1 (ZO-1) (catalog #61–7300, Invitrogen, Carlsbad, CA) overnight at 4 °C. For the second antibody, incubation with donkey anti-rabbit-Alexa Fluor 488 (1:500, catalog # ab150073, Abcam, Cambridge, UK) was performed for 30 min at room temperature. Immunoreactivity for ZO-1 was observed using confocal laser scanning microscope images (LSM700, Carl Zeiss Meditec, Oberkochen, Germany). The fluorescence intensity of the cells was measured using ImageJ software (version 1.54), and the mean intensity was calculated from three different areas of the glass slide in each sample. The experiments were conducted in triplicate.

### Statistical analyses

Unpaired students’-tests (one-tailed or two-tailed) were used for all statistical analyses. *P* values less than 0.05 were considered statistically significant.

### Supplementary Information

Supplementary Information.

## Data Availability

The data that support the findings of this study are available from the corresponding author, [SH], upon reasonable request.
