# Supplementary material for: Increased sodium fluorescein transport by corticosteroids is inhibited by a LAT-1 specific inhibitor in retinal pigment epithelial cells in vitro
Source: Sci Rep. 2023 Dec 27;13:22981. doi: 10.1038/s41598-023-50196-z (PMC10752866; doi:10.1038/s41598-023-50196-z)
Supplement: Supplementary file 1 — Supplementary Information. [file 41598_2023_50196_MOESM1_ESM.pdf]

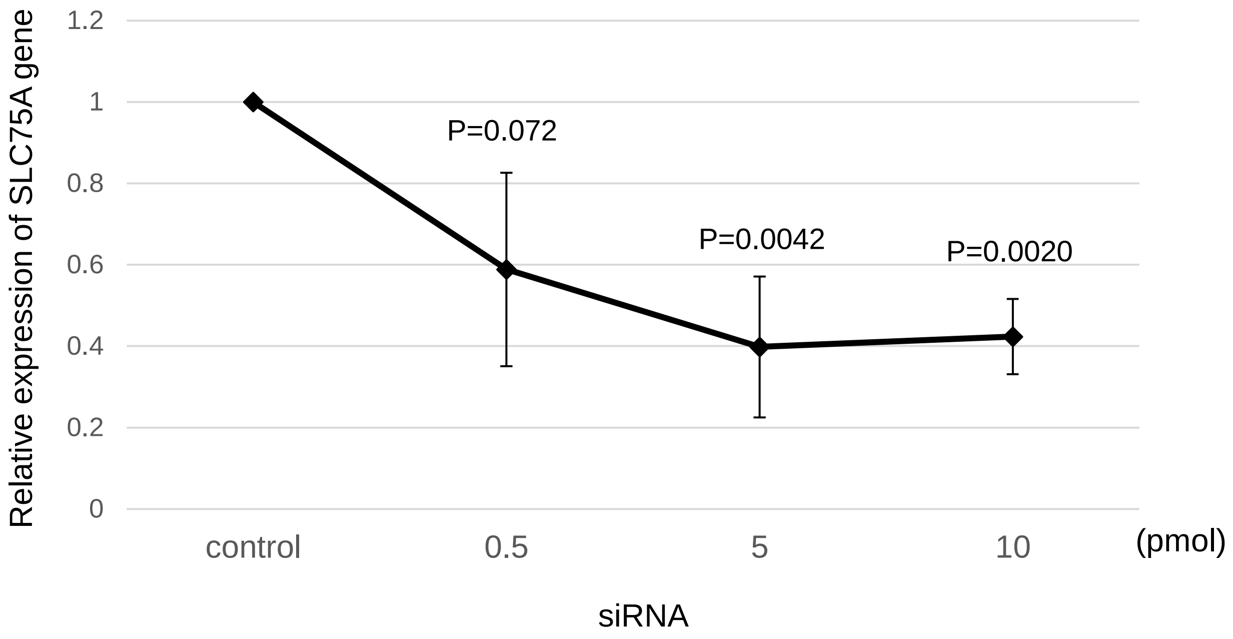

Supplementary Fig. 1. The expression of the *SLC7A5* gene in ARPE-19 cells cultured with siRNA for *SLC7A5*. Cells were grown to achieve a confluent state. The serum was withdrawn and cells were further incubated with or without siRNA for 24 hours. The real-time PCRs were performed. The amount of targeted gene expressed was normalized to an endogenous reference, GAPDH. All measurements were repeated three times and all experiments were conducted in triplicate. Values are presented as means ± SEMs.

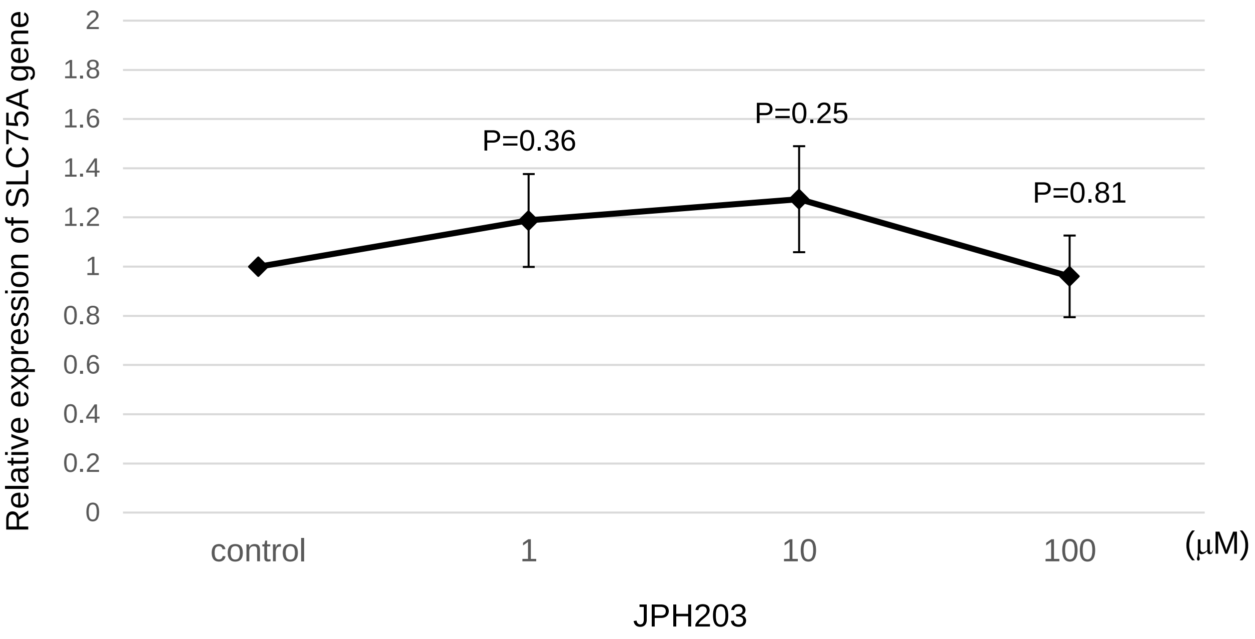

Supplementary Fig. 2. The expression of the *SLC7A5* gene in ARPE-19 cells cultured with JPH203. Cells were grown to achieve a confluent state. The serum was withdrawn and cells were further incubated with or without JPH203 for 12 hours. The real-time PCRs were performed. The amount of targeted gene expressed was normalized to an endogenous reference, GAPDH. All measurements were repeated three times and all experiments were conducted in triplicate. Values are presented as means ± SEMs.
